# Insufficient early detection of peripheral neurovasculopathy and associated factors in rural diabetes residents of Taiwan: a cross-sectional study

**DOI:** 10.1186/1472-6823-14-89

**Published:** 2014-11-24

**Authors:** Chia-Mou Lee, Chang-Cheng Chang, Mei-Yu Pan, Chyong-Fang Chang, Mei-Yen Chen

**Affiliations:** Department of Nursing, Min-Hwei College of Health Care Management, Tainan City, Taiwan; Division of Plastic Surgery, Chang Gung Memorial Hospital, Putz City, Chiayi County, Taiwan; College of Nursing, Chang Gung University of Science and Technology, No. 2, Chia-pu Rd. West Sec, Putz City, Chiayi County 61363 Taiwan

**Keywords:** Type 2 diabetes mellitus, Diabetic foot ulcer, Peripheral neuropathy, Peripheral vasculopathy

## Abstract

**Background:**

Diabetic peripheral neuropathy (PN) and peripheral vasculopathy (PV) are major causes of foot ulcers in patients with diabetes. The early detection of PN/PV with appropriate health counseling is the best strategy for preventing foot lesions. The objective of this study is to examine the prevalence and associated risk factors of PN/PV among rural community residents with type 2 diabetes mellitus (T2DM).

**Methods:**

This cross-sectional descriptive study was conducted in Taiwan from February to October 2012. Type 2 diabetes mellitus and health promotion scale, Michigan neuropathy screening instrument, and ankle-brachial pressure index values were measured.

**Results:**

A total of 404 (55% women) participated in and completed the program. The overall prevalence of PN and PV was 34.5 and 17.1%, respectively. The majority of the participants (90%) did not receive early PN/PV detection by health care providers. After adjustment for the potential confounding factors, multivariate analysis indicated that the factors determining PN/PV were age (P <0.001), living around sea coastal regions (P <0.001), high HbA1C level (P <0.01), and fewer regular health-promoting behaviors (P <0.01).

**Conclusion:**

There was a high prevalence of PN/PV among rural T2DM residents who received insufficient early detection. The early detection of PN/PV and provision of health education with customized health-promoting behaviors of people with diabetes in the rural community are important issues.

## Background

The increasing prevalence of type 2 diabetes mellitus (T2DM) worldwide poses a major public health challenge throughout developed and developing countries [1]. In Taiwan, T2DM affected 3.5% of the population in 2003 and 9.2% in 2010 [2], although this is a similar trend to other developed countries. However, according to Taiwan ICD-10 statistics, T2DM is the fourth most common cause of death with a standardized mortality rate of 26.9/10^5^ [2]. This is higher than those in the United States (14/10^5^), Japan (4/10^5^), the United Kingdom (5.0/10^5^), Singapore (13.4/10^5^), and Korea (21.8/10^5^) [1]. The prevalence is greater in rural areas; the crude death rate in Chiayi County is 50.9/10^5^, which is higher than that in most other cities near Taiwan.

Diabetic foot problem (DFP) is a complication of diabetes, and ~5% of patients with DFP require major amputation. Nearly 25% of diabetics will develop foot ulcers at some time in their lives [3, 4]. DFP results from neuropathy, vasculopathy, and immunopathy [5, 6]. Epidemiologically, the prevalence of peripheral neuropathy (PN) is 30–50% of the diabetic population worldwide. Because of its frequent occurrence, >60% of DFP cases are primarily due to an underlying neuropathy [5]. Loss of nerve function is associated with hyperglycemia as reflected in the mean level of glycosylated hemoglobin over time [7]. PN affects all components of the peripheral nervous system (sensory, motor, and autonomic), each of which contributes to foot ulcer development [5, 8, 9]. The touch and vibratory senses as well as temperature perception of the plantar foot are mediated by different nerves; damage can elicit an abnormal response and people will lose the protective sensation.

Along with neuropathy, peripheral arterial disease (PAD) is a common risk factor associated with foot complications, tissue perfusion, wound healing, deformity, and infection [9]. PAD, atherosclerotic occlusive disease of the arterial system distal to the aortic bifurcation, is a relatively common disorder in older people [10]. PAD is often a subclinical disease in which the patient has an ankle-brachial pressure index (ABI) <0.9 but does not experience symptoms. The possible mechanism of diabetic neurovasculopathy is related to decreased density of the myelinated fibers since hyperglycemia induces microvascular complications and nerve fiber loss or degeneration [8, 11]. DFP is a common cause of hospitalization as well as economic and quality of life burdens on families of diabetics and can lead to lower limb amputation [1]. In patients with DFP, the mortality rates increase 30–70% within 3–5 years after amputation [12–14].

Preventing DFP and its associated consequences is critical in the rural regions of Taiwan [15]. Enhancing patient foot care abilities and providing health promotion programs effectively prevent ulceration [15–17]. Through early screening that reduces risk factors and prompt treatment of PN and peripheral vasculopathy (PV), DFP can be prevented in ~85% of cases [18, 19]. Therefore, providing early detection of peripheral sensory neuropathy and the peripheral circulation is important for patients with diabetes, particularly rural elders. Non-invasive instruments including foot examinations using the Michigan neuropathy screening instrument (MNSI), ABI, and physiological indicators are cost-effective strategies [15, 20]. However, these methods are unpopular in primary healthcare. Early DFP prevention and detection is often neglected in primary care settings. Therefore, we explored the prevalence of PN and PV, access to early screening, and the associated risk factors of PN/PV among rural community residents with T2DM. Based on previous research, we hypothesized that among community residents, T2DM and abnormal PN or PV would be associated with older age, diabetic duration, poor glycemic control, and a lack of health-promoting behaviors [11, 15].

## Methods

### Sample and setting

This study was part of a longitudinal cohort study of health promotion for preventing T2DM foot ulceration among rural community residents (2010–2012) in southwestern coastal Taiwan. A cross-sectional descriptive design was conducted in 18 township health centers between February and October 2012 in Chiayi County. Purpose sampling was used from their local DM registration files, and the subjects were invited to participate by the district’s public health nurse. Selection criteria included subjects who were: (1) previously diagnosed with T2DM by a physician, (2) first-time contacts in the longitudinal cohort study, (3) able to complete the questionnaires in Mandarin and Hokkien dialects either by themselves or via interview, (5) age ≥20 years, and (6) willingness to participate. Exclusion criteria were: (1) serious mental problems, including those who received a disability certificate or diagnosis of dementia by a physician or community welfare organization, (2) serious diabetes complications, e.g., lower limb amputation, and (3) inability to walk to the local health center. Owing to the lack of a previous study reporting data on the association between health-promoting behaviors and PN or PV, we used estimated data to determine the sample size. Given that the prevalence of an unhealthy lifestyle (i.e. health-promoting behavior score <70) of the patients with and without peripheral neurovasculopathy (PNV) was 0.50 and 0.35, respectively, with a significance level of 0.05 and a statistical power of 0.80 on a two-tailed test that resulted in a minimum required sample size of 170 for each group [21].

### Instruments

The risk factors of PN are associated with diabetes duration, increased glycosylated hemoglobin level, obesity, smoking, and hypertension [7]. Five instruments and major health indicators were used to assess the physiological biomarkers and health-promoting behaviors.

1. *Demographic characteristics* were as follows: Age, sex, marital status, diabetes duration, body height, body weight, education level, occupation, current medications (oral antidiabetic drugs [OAD], insulin, or both prescribed by a physician), foot examination status (received screening tests for peripheral neurological and vascular assessment by healthcare providers before the launch of this community screening program), past health history, smoking habits, and glycemic control (including diet and exercise for at least 20 min/day or 150 min/week).
2. *Peripheral neurological assessment* was measured by the MNSI (a total score of 0–10 was obtained). The MNSI is a reliable and valid scale for detecting diabetic neuropathy [15, 22] specifically for the touch-pressure sensation test using a 5.07/10 g Semmes–Weinstein monofilament applied perpendicularly to test sites on the feet. Vibration perception threshold testing––a semi-quantitative assessment of vibration sensation––was conducted with a 128-Hz turning fork on the dorsum of the big toe and is also important in detecting the early symptoms of neuropathy [15, 23]. The investigators and research assistants assessed each participant’s feet against five parameters: (a) appearance (inspection of lower limbs for deformity); (b) ulceration status; (c) vibration sensation on the dorsum of the big toe; (d) ankle reflexes; (e) and touch-pressure sensation. Each received 1, 0.5, or 0 points. PN was diagnosed when a patient had an MNSI score ≥2.5 on a 10-point scale.
3. *Peripheral vascular assessment:* ABI was calculated from ankle/arm pressure using the Cardio-Vision Model (MS-2000; Mars Medical Products Co., Ltd., San Chung City, Taipei, Taiwan). ABI values were classified as follows: ≥0.90 normal and ≤0.89 was defined as PV. However, an ABI >1.3, seen as poorly compressible, might be due to medial calcification and cause a false negative result [10, 12].
4. *Diabetes-related physiological indicators:* (A) The most recent fasting blood glucose (FBG) and glycohemoglobin (HbA1C) values were used; the results obtained from the diabetes records for each subject were saved on a computer in the local health center. (B) Waist circumference (WC) was measured during the study and used to assess central obesity by measurement of the mid-abdominal distance between the last rib margin and the iliac crest. Normal values were defined as FBG <130 mg/dL, HbA1C <7.0%, WC <80 cm for women, and WC <90 cm for men [24].
5. *The T2DM and health promoting scale* (T2DHP) was used to measure the health-related behaviors of patients with diabetes. The T2DHP included 28 questions designed to determine the frequency of health-promoting behavior [25]. The questionnaire used a five-point response format to obtain data regarding the frequency of reported behaviors (never, rarely, sometimes, usually, or always), with ratings of 1–5. A simplified version of the T2DHP comprised six dimensions of behavior: physical activity (seven items, e.g., I do moderate-intensity exercise for >150 min every week, I exercise indoors in bad weather), risk reduction (seven items, e.g., I check my toes and feet for wounds, I brush my teeth after meals), stress management (five items, e.g., I try to relax when I am in a bad mood), enjoyment of life (three items, e.g., I arrange my daily life well), health responsibility (three items, e.g., I visit a doctor periodically), and a healthy diet (three items, e.g. I avoid eating foods high in fat, I eat a balanced diet every day). The reliability coefficient for the total scale was 0.89, while the alpha coefficients for the subscales were 0.63–0.86. Concurrent validity indicated that the T2DHP was significantly positively associated with diabetes control. Construct validity was established using factor analysis. T2DHP was proven as a reliable and valid tool for assessing patients with T2DM and might predict glycemic control [25, 26].

### Procedure and ethical considerations

This study was conducted after the Ethical Committee of the Institutional Review Board (Chang-Gung Memorial Hospital Ethics Committee 100-4628B). Two weeks before study initiation, each participant received a questionnaire with a cover letter emphasizing confidentiality; each questionnaire was filled out anonymously. The written informed consent was obtained from each participant by public health nurses after the purpose and procedure had been explained. Data were collected during interviews by research assistants with registered nursing licenses. During the data analysis process, confidentiality was maintained by data coding to prevent personal identification. All of the data were stored in a locked cupboard by the primary investigator.

### Analysis

The data were analyzed using SPSS 17 statistical software (SPSS Inc., Chicago, IL, USA). All tests were two-sided and values of P <0.05 were considered statistically significant. Participants who tested positive for PN or PV were recorded as being in the PNV group. The t-test and chi-square method for testing the mean difference and equality of proportions were used to compare the personal factors or health-promoting behavior with or without PNV. To investigate the potentially determinant factors associated with PNV, the variables that were significant (*P* <0.05) on previous univariate analyses (t-test or chi-square test) were further introduced into a multivariable logistic regression analysis with forward stepwise selection.

## Results

A total of 428 participants were enrolled. Twenty-four subjects failed to complete the questionnaires, so 404 subjects ultimately provided valid data. Participants were 28–90 years of age, and 66% were >65 years. The mean diabetes duration was 8.3 years. The majority of participants (82%) completed primary school or less. More than half of the participants lived around the plains (55%). About 64% were retired or unemployed. Nearly 90% of participants reported that they regularly took medication; 94% were taking OAD and a few were taking insulin. In addition, 40.6% and 51.0% did not have a healthy diet or exercise regularly to control their blood sugar (Table 1).

**Table 1:** **Demographic characteristics (N = 404)**

Variables	N (%)
Gender	
Male	181 (44.8)
Female	223 (55.2)
Age(years)	
< 65	136 (33.7)
≥ 65	268 (66.3)
Educational levels	
≤ Primary school	331 (81.9)
≥ Secondary school	73 (18.1)
Area of residence	
Plain region	224 (55.4)
Sea coastal region	67 (16.6)
Mountainous region	113 (28.0)
Regular taking medication	
Yes	363 (89.9)
No	41 (10.1)
Glycemic control*	
Only OAD	377 (94.0)
OAD combined insulin	24 ( 6.0)
Glycemic control-diet	
Yes	240 (59.4)
No	164 (40.6)
Glycemic control-exercise	
Yes	198 (49.0)
No	206 (51.0)
Systolic blood pressure (mmHg)	
Normal (≤130 mmHg)	121 (30.0)
Abnormal (>130 mmHg)	283 (70.0)
Diastolic blood pressure (mmHg)	
Normal (≤80 mmHg)	236 (58.4)
Abnormal (>80 mmHg)	168 (41.6)
Fasting blood glucose (mg/dl)*	
Normal (<130)	141 (39.7)
Abnormal (≥130)	214 (60.3)
HbA1c*	
Normal (<7%)	117 (32.9)
Abnormal (≥7%)	238 (67.1)
Waist circumference (cm)	
Normal (male ≤ 90, female ≤ 80)	121 (30.0)
Obesity (male > 90, female > 80)	283 (70.0)
Body mass index (BMI)	
Normal (<24)	98 (24.2)
Overweight (24 ~ 27)	147 (36.4)
Obesity (>27)	159 (39.4)
Have you received foot examination before?	
Yes	40 (9.9)
No	384 (90.1)
Smoking habit^#^	
Current users and cessation	105 (26.0)
Never user	299 (74.0)
Michigan neuropathy screening index*	
≤2.0	247 (65.5)
≥2.5	130 (34.5)
Ankle brachial pressure index (ABI)	
Normal (0.9 ~ 1.3)	335 (82.9)
Abnormal (<0.9 & >1.3)**	69 (17.1)

*With missing data; OAD: oral anti-diabetes drugs; ^#^Smoking habit: Male; N = 99 (94.3%), female; N = 6 (5.7%); ** ABI > 1.3, N = 14 (3.5%).

Participants with an abnormal systolic blood pressure, abnormal FBG level, abnormal HbA1c level, high WC, or who were overweight or obese consisted of 70%, 60%, 67%, 70%, and 76% of the population, respectively. Almost all of the participants (90%) reported that they had not received a foot examination by a healthcare provider prior to the screening program, while 26% of the participants said they were current smokers or had just quit during the past year. Excluding the missing data, Table 1 shows that 34.5% of participants had PN and 17.1% had PV, whereas 3.5% had an abnormal ABI of >1.3 and 12.4% had both PN and PV. Since participants who tested positive for PN or PV were considered positive for PNV in this study, the overall prevalence of PNV was 46.5%. Tables 2 and 3 show that participants living in the coastal region (χ^2^ = 25.68, P <0.001), with older age (t = -4.86, P <0.001), higher FBG (t = -2.60, P =0.01), and higher HbA1c (t = -2.16, P =0.03) tended to have a high frequency of PNV.

**Table 2:** **Univariate analysis of factors associated with PNV**

	PNV			
Variables	No N (%)	Yes N (%)	χ ^2^	***P***
Gender				
Female	114 (57.3%)	99 (52.7%)	0.84	.36
Male	85 (42.7%)	89 (47.3%)		
Educational levels				
≤Primary	143 (77.7%)	147 (84.5%)	2.66	.10
≥Secondary	41 (22.3%)	27 (15.5%)		
Area of residence				
Plain region	58 (29.1%)	49 (26.1%)	25.68	<.001
Sea coastal region	126 (63.3%)	89 (47.3%)		
Mountainous region	15 (7.5%)	50 (26.6%)		
Regular taking medication				
No	13 (6.8%)	14 (7.7%)	0.13	.72
Yes	179 (93.2%)	167 (92.3%)		
Smoking habit			0.01	.94
Current users and cessation	52 (26.3%)	50 (26.6%)		
Never user	146 (73.7%)	138 (73.4%)		
Glycemic control- diet				
No	74 (37.4%)	82 (44.1%)	1.79	.18
Yes	124 (62.6%)	104 (55.9%)		
Glycemic control- exercise				
No	90 (45.5%)	105 (56.8%)	4.89	.03
Yes	108 (54.5%)	80 (43.2%)

**Table 3:** **Univariate analysis of factors associated with PNV**

	PNV			
Variables	No Mean (SD)	Yes Mean (SD)	t	***P***
Age (years)	66.2 (8.6)	70.7 (9.3)	-4.86	<.001
Duration of Diabetes (years)	7.9 (6.6)	8.4 (7.4)	-0.67	.51
Systolic blood pressure (mmHg)	139 (17)	140 (19)	-0.58	.56
Diastolic blood pressure (mmHg)	79 (10)	78 (9)	0.76	.45
Fasting blood sugar (mg/dl)	143 (41)	158 (61)	-2.60	.01
HbA1c (%)	7.7 (1.5)	8.1 (1.8)	-2.16	.03
Body mass index	26.6 (4.1)	26.4 (3.9)	0.53	.60
Waist circumference (cm)	89.9 (10.1)	90.2 (9.6)	-0.28	.78
T2DHP- Exercise	21.6 (08.0)	19.8 (7.3)	2.30	.02
T2DHP- Reduce risk	18.6 (06.7)	16.6 (6.0)	3.06	<.01
T2DHP- Life	16.6 (03.2)	15.4 (4.0)	3.07	<.01
T2DHP- Stress	17.2 (02.6)	16.6 (3.3)	2.32	.02
T2DHP- Responsibility	12.9 (02.4)	12.1 (2.9)	3.21	<.01
T2DHP- Healthy diet	19.6 (04.4)	18.5 (4.6)	2.32	.02
T2DHP total score	106 (18)	99 (18)	4.07	<.001

PNV-positive participants engaged in fewer health-promoting behaviors, e.g., regular exercise (t = 2.30, P = 0.02) and were less likely to actively reduce their risk factors (t = 3.06, P <0.01), enjoy life (t = 3.07, P <0.01), use stress management tools (t = 2.32, P = 0.02), take responsibility for their health (t = 3.21, P <0.01), or adopt a healthy diet (t = 2.32, P = 0.02), and they had a high total T2DHP score (t = 4.07, P <0.001) (Table 3). After adjustment for potential confounders including sex, smoking habits, age, obesity, and FBG, the logistic regression model (Table 4) indicates that older patients (odds ratio [OR] = 2.86, P <0.001) and those living in a coastal region (OR = 4.06, P <0.001) were more likely to be PNV-positive than those <65 years of age who lived in mountainous areas. Each of these patients had an increased HbA1c (P <0.01) and were less likely to adopt fewer health-promoting behaviors (P = 0.03), both significant predictors of PNV.

**Table 4:** **Logistic regression analysis of determinant factors on PNV**

Variables	B	S.E.	Odds ratio	***P***	95% CI
Age (years)					
≥ 65	1.05	0.27	2.86	<.001	1.69 ~ 4.83
< 65^*^					
Area of residence					
Mountainous region	- 0.02	0.27	0.98	.93	0.57 ~ 1.67
Sea coastal region	1.40	0.35	4.06	<.001	2.05 ~ 8.03
Plain region^*^					
HbA1c (%)	0.22	0.08	1.25	<.01	1.08 ~ 1.45
T2DHP total score	- 0.02	0.01	0.99	.03	0.97 ~ 0.99

^*^Reference group.

## Discussion

Two key findings emerged from this study. First, a high prevalence of PN (34.5%), PV (17.1%), and poor glycemic control occurred in rural community residents with T2DM. Second, the use of non-invasive instruments in the early detection of PN/PV was neglected by many primary healthcare providers.

### Early detection of PNV is necessary for and important to preventing diabetic foot ulcerations

Diabetic PN/PV increases the risk of diabetic foot complications [3, 9]. Using the cut-off score of MNSI ≥2.5 for this current study, we found that the incidence of PN and PV was 34.5% and 17.1%, respectively. Compared to a similar study in Taiwan using the same instrument, Chen et al. [11] used MNSI ≥3 as the cut-off point for PN and showed a 22.2% prevalence of PN in rural areas. However, in Europe, Geerts et al. [27] found a higher prevalence of PN at 43.2–57.5%. Regarding the prevalence of PV, the present study revealed values that were similar to the Asian values and lower than those in the United States [28] but higher than those in the other study in Taiwan. According to Rhee et al. [28] and Tseng et al. [29], the prevalence of PV in Taiwan, Asia, and the United States was 10.0%, 17.7%, and 20%, respectively. The reasons for these differences might be due to the samples in our study being taken from the rural community rather than from hospitals. Another reason might due to instrument limitations because 3.5% of patients with abnormal ABI values were categorized as having arterial calcification. The alternative use of the toe-brachial index (TBI) may give a better indication of the PAD extent in the distal extremities because the arteries within the digits are less affected by calcinosis. William et al. [14] demonstrated that TBI was a more effective screening method than ABI, but TBI was not used here since it is not affordable and was not available for community research. Despite ABI being a feasible assessment tool in primary care settings, its effectiveness for detecting PAD in community screenings leads to higher false negatives in patients with diabetes, which must be further considered.

However, when we asked the participants "have you received foot examination in recent years in which you took off your shoes and a healthcare provider examined your blood pressure and checked for numbness in your feet", 90% said they had not undergone these examinations as far as their physicians or nurses could remember. Many studies indicated that educating diabetes patients about proper foot care and conducting periodic foot examinations decreases the prevalence of ulceration [6, 17, 20]. One study focused on 49 foot ulceration patients around the sea coastal region in Taiwan; Li et al. [6] mentioned that "the devil is in the details" and reported that all (100%) farmer and fisherman participants with diabetic foot ulceration suffered from PN. Like Li et al. [6], we showed that PNV was four times more prevalent in individuals with diabetes living around the sea coastal region than in individuals with diabetes living in the plains and mountains, which might due to socioeconomic disadvantages since the majority of people in the western coastal region tend to have lower incomes and less education and access to healthcare. Therefore, our health sector, especially in terms of community nurses in the diabetes share-care systems located around the sea coastal region, needs to provide early detection of PN/PV using simple and non-invasive screening tools in primary healthcare settings.

### Improving the lifestyles of diabetic residents

Nearly 90% of the participants took their medication regularly and 59% had chosen a suitable diet for their diabetes control. These findings show that glycemic control and health status in many of the participants were under the recommended standard set by diabetes experts. It is surprising that most elderly diabetic residents reported regularly taking medication in their daily life but had poor biochemical data. The reasons for the discrepancy might be due to data collection and recall bias since we asked "do you regularly take diabetes medication" but did not check their medication box or ask their family members for details, such as medication doses, times taken, and other drug amounts. It is necessary to explore the limitations and ability for rural residents with diabetes to receive more ideal glycemic control.

Most participants with PN or PV living in the western coastal region did not have a healthy lifestyle, e.g., exercise regularly, display risk-reducing behaviors, take responsibility for their health, or choose a healthy diet. Some evidence indicates that engaging in health-promoting behavior is positively associated with decreasing FBG and HbA1c levels [25]. Lowering blood sugar levels and blood pressure markedly reduce diabetes complications, including the risk of amputation [11]. According to a longitudinal community-based study, Chen et al. [11] found that implementing community health promotion programs was beneficial for rural diabetics in terms of relevant physiological parameters, such as a reduced body mass index, WC, FBG, and MNSI and increased ABI in both legs. In examining the misconception among rural residents with diabetes, Huang et al. [30] showed that the phenomenon of "I feel good, but the data says no" could be found in many residents with diabetes living in disadvantaged areas. Further studies should consider the perspective of health literacy and provide customized health-promoting behaviors for the diabetic population in rural communities, which are important issues.

### Study limitations

This study has its limitations. First, selection and recall bias must be considered because all of the participants had different durations of diabetes and other health conditions. This might limit our understanding of the associated risk factors between PNV and rural setting in individuals with diabetes. Second, the samples were not entirely random, and most of the participants had lower socioeconomic status, i.e., had completed <6 years of education or were elderly. This will limit any generalization of these findings and be a potential threat to the internal validity of the health literacy issue, which requires consideration. Third, the measurement tools have some limitations, such as a low ABI being taken as a good marker for PAD, and we used ABI 0.9–1.3 as the normal range. Finally, the elevated false negative diagnosis rate of diabetes due to medial calcinosis was not considered in this study.

## Conclusions

Despite some limitations, these findings show: (1) a high prevalence of PN/PV and poor standard of glycemic control, and (2) insufficient early detection of diabetic foot ulceration using simple and non-invasive assessment instruments by community healthcare providers among rural community residents with T2DM. The risk factors associated with PNV were older age, living in a sea coastal region, having a higher HbA1c, and the lack of health-promoting behaviors. These findings highlight the need to include the early detection of PNV and measure the health-promoting behavior in rural regions to more fully understand the factors that might predict the development of foot ulcers in individuals with T2DM.

Although 99% of the Taiwanese population is covered by the national health insurance, it cannot be taken for granted that all people with diabetes receive the same professional or quality service as those in urban areas. Therefore, we advocate eliminating this inequity and taking action to influence the policies for these disadvantaged people. We also suggest that future studies should focus on the continuation of changes of PN, PV, and glycemic control among rural community residents with T2DM. Furthermore, we advocate the important strategies of developing culturally competent educational interventions (especially for those with little education for whom educational materials, e.g., media and pamphlets, should be adapted) to improve neurovascular function in farmers and fishermen with T2DM.
